# Sea-surface *p*CO_2_ maps for the Bay of Bengal based on advanced machine learning algorithms

**DOI:** 10.1038/s41597-024-03236-w

**Published:** 2024-04-13

**Authors:** A.P. Joshi, Prasanna Kanti Ghoshal, Kunal Chakraborty, V. V. S. S. Sarma

**Affiliations:** 1 Indian National Centre for Ocean Information Services, Ministry of Earth Sciences, Hyderabad, India; 2 Faculty of Ocean Science and Technology, Kerala University of Fisheries and Ocean Studies, Kochi, India; 3 CSIR-National Institute of Oceanography, Visakhapatnam, India

**Keywords:** Ocean sciences, Carbon cycle

## Abstract

Lack of sufficient observations has been an impediment for understanding the spatial and temporal variability of sea-surface *p*CO_2_ for the Bay of Bengal (BoB). The limited number of observations into existing machine learning (ML) products from BoB often results in high prediction errors. This study develops climatological sea-surface *p*CO_2_ maps using a significant number of open and coastal ocean observations of *p*CO_2_ and associated variables regulating *p*CO_2_ variability in BoB. We employ four advanced ML algorithms to predict *p*CO_2_. We use the best ML model to produce a high-resolution climatological product (INCOIS-ReML). The comparison of INCOIS-ReML *p*CO_2_ with RAMA buoy-based sea-surface *p*CO_2_ observations indicates INCOIS-ReML’s satisfactory performance. Further, the comparison of INCOIS-ReML *p*CO_2_ with existing ML products establishes the superiority of INCOIS-ReML. The high-resolution INCOIS-ReML greatly captures the spatial variability of *p*CO_2_ and associated air-sea CO_2_ flux compared to other ML products in the coastal BoB and the northern BoB.

## Background & Summary

Oceans play a significant role in regulating the amount of CO_2_ in the atmosphere. Human-induced anthropogenic activities have increased atmospheric CO_2_, counterbalanced by the increasing global ocean CO_2_ uptake. Thus, the oceans become over-saturated, and as a result, the regional oceans have been increasingly becoming sources of atmospheric CO_2_. An increase in ocean sink strength has been seen in the past decade ($\approx 2.5\pm 0.6$ GtC per year^1^). The first two years of this decade are reported to have even higher ocean sink strength ($\approx 3.0\pm 0.6$ GtC per year in 2020^2^ and $\approx 2.9\pm 0.6$ GtC per year in 2021^3^). Based on previous literature, the estimated ocean sink strength of the global coasts has decreased to $\approx 0.2$ PgC per year^4,5^. On the other hand, current research shows an increase in the continental shelves' sink strength^6^. The wintertime CO_2_ sink in the northern South China Sea behaves stronger after 2007, although this sea area still serves as a weak annual source of atmospheric CO_2_^7–9^. These global studies have highlighted the importance and role of the ocean in modulating the atmospheric CO_2_, and hence the environment. With the rise in importance of studying the sea-surface partial pressure of CO_2_ (*p*CO_2_), the paucity of measured data (especially on a regional scale) is an impediment for observational analysis and model validation^10–12^.

This study aims to develop *p*CO_2_ climatological data based on observation for the Bay of Bengal region (BoB). The BoB is recognized for having complex physical dynamics because of significant freshwater input and its distinctive geographical location. The Ganges-Brahmaputra river system, the second largest river system in the world, brings in high freshwater along with organic pollutants into the BoB region^13,14^. The freshwater influx increases stratification and reduces the vertical mixing (thick barrier layer), which influences the absorption and/or outgassing of atmospheric CO_2_ in BoB^15^. The nutrients brought down by these rivers decrease the ocean-surface *p*CO_2_ in the offshore region, but its influence diminishes away from the coast^14^.

The BoB is influenced by the seasonal reversing coastal currents (East India Coastal Currents (EICC)). From February to March, the EICC brings high saline waters from south to north, which weakens stratification and initiates upwelling. The upwelling brings high subsurface dissolved inorganic carbon (DIC) to the surface, which increases the sea-surface *p*CO_2_^16,17^. The EICC flows south from October to December, carrying less saline waters from the north towards the south. This results in low sea-surface *p*CO_2_ (≈ 320 *μ* atm) values during this period. The freshwater plume spread due to this southward motion of EICC results in low sea-surface *p*CO_2_ values in the northern BoB^15^. The spatial pattern of the sea-surface *p*CO_2_ is dominated by the biological and thermal mechanisms^14,18^. Temporal evolution is dominated by solubility, primarily increased by sea-surface temperature (SST) and decreased by DIC^18,19^.

The sparse observations of sea-surface *p*CO_2_ constitute a significant hindrance in validating the coupled bio-physical model simulated ocean carbon cycle. The studies^15,17–20^ based on bio-physical models often validate the model with the BOBOA (Bay of Bengal Ocean Acidification) mooring^21^ at 15° N, 90° E. Another popular observation data is the SOCAT (Surface Ocean Carbon-dioxide Surface Atlas) data^22^, which has poor spatial and temporal coverage in the BoB region. These models are often compared with observation-based products like GLODAP^23^, which is a spatial annual mean data, and Takahashi data^24^, which has a very coarse resolution (4*°* × 5*°*). The observation-based products suffer due to a lack of observations in the BoB region, specifically, the unavailability of data near the coast^25,26^. The high freshwater flux, affecting the physical dynamics, also affects these observation-based products as the general assumption (e.g., failure of linear relation assumption of potential alkalinity and sea-surface salinity (SSS)) often fails in the BoB^24^.

Besides bio-physical models, the use of regression models^27–29^ is popular to understand the carbonate dynamics of the BoB region. These regression models emulate the sea-surface *p*CO_2_ with relatively larger errors. The linearity assumption between the dependent and independent variables is not always true. Region-specific Machine Learning (ReML) algorithms showed promising results for the central BoB^30^. Hence, this study attempts to construct spatiotemporal sea-surface *p*CO_2_ maps for the BoB using observations and advanced ML techniques.

## Methods

**Fig. 1:**
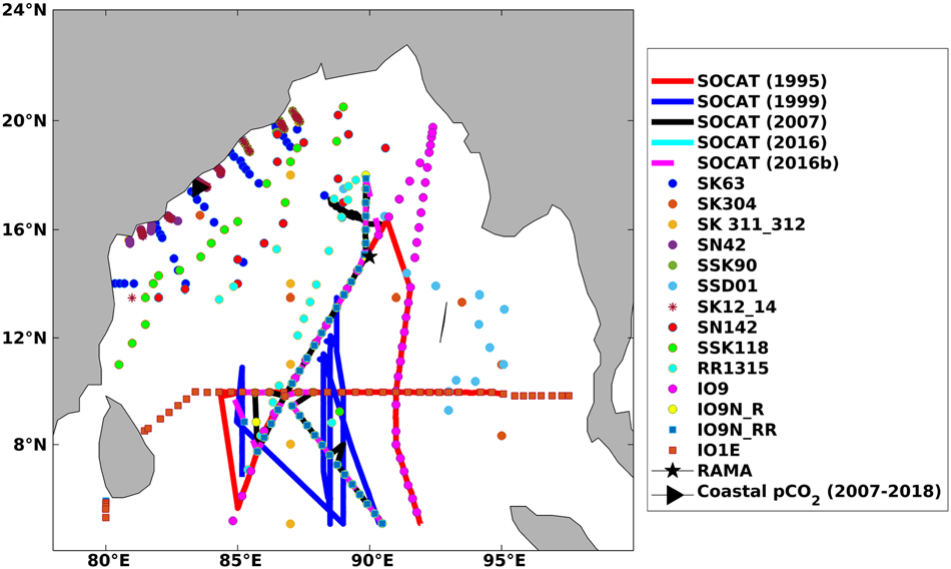
Representation of the study region (BoB) and the *available of p*CO_2_ observations included in this study.

This study includes a significant number of open and coastal ocean *p*CO_2_ observations and associated variables regulating *p*CO_2_ variability in BoB to come up with a data set that could aid in training advanced ML models (Fig. 1). We assume that the sea-surface *p*CO_2_ is a function of sea-surface temperature (SST), sea-surface salinity (SSS), mixed layer depth (MLD), atmospheric CO_2_ mole fraction (xCO_2_) and chlorophyll-a (CHL). The influence of the above-mentioned independent variables in regulating sea-surface *p*CO_2_ variability has been included as a proxy of different mechanisms (thermal, solubility, mixing, air-sea interaction, and biology).

### Data acquisition

**Fig. 2:**
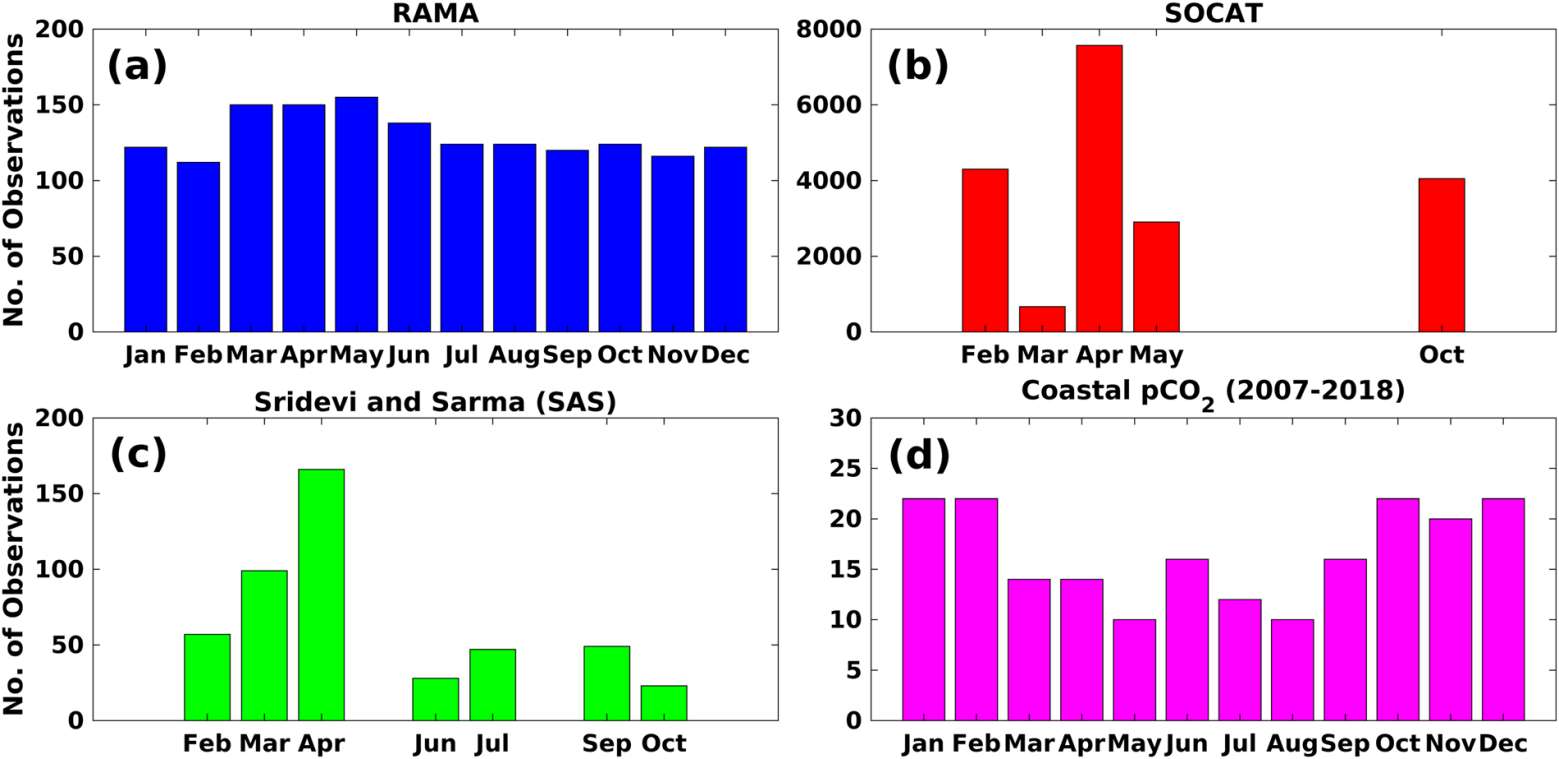
Monthly observations of SST, SSS, and *p*CO_2_ were acquired from various sources. The RAMA buoy (**a**) provides the sea-surface *p*CO_2_ observations between November 2013 to December 2018. All ship-based observations (available in the SOCAT database) from 1991 to 2020 were used in this study (**b**). Further, additional ship-based observations available from 1991 to 2019 (denoted here as SAS data) were also included (**c**). The availability of *p*CO_2_ data from coastal transects from 2007 to 2018 is shown in (**d**).

SST and SSS observations, along with collocated sea-surface *p*CO_2_, are available at the locations shown in Fig. 1. We obtain the synthesised SST, SSS, and *p*CO_2_ observations from SOCAT (https://www.socat.info/index.php/data-access/)^22^ and other locations shown in Fig. 1. Other than the observations at SOCAT and RAMA buoy locations, the available observations are addressed here as SAS (Sridevi and Sarma) data^28^. The data collection and quality control methods are elaborately available in the literature corresponding to each of these data^22,28^. The monthly data frequency of collocated SST, SSS, and *p*CO_2_ from various sources is shown in Fig. 2. The maximum number of observations is sourced from SOCAT (Fig. 2b), but it does not uniformly cover all the months. Specifically, in the open ocean SOCAT and SAS data, the observations are unavailable for the winter monsoon season (Dec, Jan, Feb). But these data provide a very good spatial coverage in other seasons. Further, the winter monsoon season observations are available from two sources: firstly, from the RAMA mooring and secondly, from the coastal transects of SAS data (as shown in Fig. 1). All ship-based observations (available in the SOCAT database) from 1991 to 2020 were acquired for this study. In the SAS data, the observations were available from 1991 to 2019.

CHL concentration is not available in SOCAT and SAS (except in a few locations) database; hence, we use a merged satellite product OC-CCI (Ocean Color Climate Change Initiative, available at https://climate.esa.int/en/projects/ocean-colour/data/)^31^. This data has excellent spatial (1/12°) and temporal coverage (1997–2020). We extract collocated monthly CHL concentrations from OC-CCI at the available observation locations. Like CHL, MLD data cannot be obtained from SOCAT and SAS since temperature and salinity depth profiles are unavailable. So we obtain MLD from GLORYS12V1 product, which is a CMEMS eddy-resolving reanalysis product (data available at https://data.marine.copernicus.eu/product/GLOBAL_MULTIYEAR_PHY_001_030/download). The MLD product has a spatial resolution of 1/12°, and the data is available from 1993–2020. The xCO_2_ is obtained from CAMS CO_2_ atmospheric inversion product^32–34^ (https://atmosphere.copernicus.eu/). The xCO_2_ data has spatial coverage of 0.25° and is available from 1985–2020. We use the nearest-neighbor interpolation method to find collocated data at the available sea-surface *p*CO_2_ observation locations.

We checked the data distribution before using these data for training and predictions. The MLD and CHL data are converted to normal by taking their log transformation. Since ML models are known to be sensitive to outliers (> 3 *σ*), these are removed from the available data.

### Splitting and scaling data

To avoid data leakage, using a train-test split from the Scikit-Learn module, we divided the data into train-set (80%) and test-set (20%)^35^. The same training and test data are used for all ML models used in this study, which gives an advantage in testing model performance with respect to common test data. The K-fold (10 K-folds) technique is utilized for training each model, which aids in circumventing the over-training issue.

The data is then scaled using the StandardScaler method from the pre-processing libraries of the scikit learn. Scaling converts all the data between the range −1 to 1 with a mean of zero and a standard deviation of one. This process, called standardization, simplifies learning new things for ML models.

### Models

The study tests four advanced ML algorithms, and the best among the four is used to create sea-surface *p*CO_2_ maps for the BoB. The description of each of these algorithms is as follows:

- **Multiple Linear Regression (MLR)**
  Multiple linear regression is an analysis that builds the output variables from the input variables. The approach attempts to link the response and interpretation variables linearly. It extends the traditional least square strategy because it considers numerous pertinent variables.
  The use of multiple linear regression is evident and well-established for different applications in the literature^27–29^. It is to be noted that advanced ML can only be used if a significant number of observations are available. The multiple linear regression equation to predict sea-surface *p*CO_2_ is as follows:
  1 $$p{{\rm{C}}{\rm{O}}}_{2}=365.94+11.92\times {\rm{S}}{\rm{S}}{\rm{T}}+7.45\times {\rm{S}}{\rm{S}}{\rm{S}}-1.23\times {\rm{l}}{\rm{o}}{\rm{g}}({\rm{C}}{\rm{H}}{\rm{L}})+0.86\times {\rm{l}}{\rm{o}}{\rm{g}}({\rm{M}}{\rm{L}}{\rm{D}})+19.29\times {{\rm{x}}{\rm{C}}{\rm{O}}}_{2}$$
- **Artificial Neural Network (ANN)**
  The Artificial Neural Network (ANN) is a part of artificial intelligence based on the biological neural system. It has become common practise to establish the *p*CO_2_ for regional scales^30,36–39^. The ANN comprises interconnected neurons that interpret incoming data like how the human brain learns. Each connection’s signals are absolute values, and each neuron’s output is calculated as the sum of its inputs, a nonlinear function. The edges are another name for the physical link that exists between neurons. Weights are allocated to the neurons and edges, and they self-adjust to get the best results. An input, an output, and at least one hidden layer compose an ANN. The neurons in the input layer equal the number of input parameters (independent variables) as the input layer is linked to the input data. Similarly, the output layer’s neurons match the number of dependent variables. A signal can go through numerous hidden layers comprising several neurons, from the input to the output layer. The hidden layer’s main objective is to establish a link between the output and input variables.
  The ANN hyper-parameters are tuned using KerasTuner^40^ class from the Keras library. Rectified Linear Unit (ReLU)^41^ activation function is used for the hidden layers and the Linear activation function for the output layer. The network is optimized using the Adam optimizer^42^. The loss function, Mean Absolute Error, is employed and must be minimized. Two executions per trial are allowed with the parameters set for 100 trials.
  There are 18 hidden layers in the optimized ANN used in this study. Table 1 displays the neurons associated with each hidden layer. The model operates most well at a 0.0001 learning rate.

**Table 1:** Neurons in each hidden layer.

Hidden Layer	Number of neurons
Layer 1	32
Layer 2	24
Layer 3	64
Layer 4	44
Layer 5	80
Layer 6	24
Layer 7	22
Layer 8	30
Layer 9	42
Layer 10	78
Layer 11	34
Layer 12	72
Layer 13	38
Layer 14	72
Layer 15	50
Layer 16	62
Layer 17	46
Layer 18	26
- **Xtreme Gradient Boosting (XGB)**
  Extreme Gradient Boosting (XGB)^43^ is one of the members of the family of boosting algorithms built on decision trees. The gradient-boosted algorithm’s performance and computational speed were both expanded to produce the XGBoost algorithm. Since it performed well for the central BoB region^30^, the model’s great speed and accuracy motivate us to compare its performance to that of other models. Only the residuals are supplied to the following weaker learners once the trees or vulnerable learners have been added in sequential order. This method helps to cut down on errors. Contrary to gradient descent, the Newton boosting based on the Newton Raphson method accelerates the approach to global minima.
  Similar to the ANN, the XGBoost model also has tunable hyperparameters. Following previous literature^30^, we employ the Optuna optimization framework^44^ to fine-tune the hyper-parameters. At https://xgboost.readthedocs.io/en/stable/parameter.html one may find the description for each of the XGB hyper-parameters. The hyper-parameters range and final optimized values are shown in Table 2.

**Table 2:** Optimized values of the XGB hyper-parameters.

Hyper-parameters	Range or Options	Optimized Value
lambda	0–1.0	0.8634
alpha	0-1	0.2574
subsample	0-1	0.6920
Booster	gbtree/gblinear/dart	gbtree
colsample_bytree	0-1	0.6460
max_depth	10–100 (step = 1)	93
min_child_weight	1–100	36
learning_rate	1 × e-08-1	0.0001
gamma	1 × e-08-1	5.546 × e-07
n_estimators	100–150 (step = 1)	131
grow_policy	depthwise/lossguide	lossguide
- **Random Forest(RF)**

As XGB belongs to a family of boosting algorithms, Random Forest (RF)^45^ belongs to a family of bagging algorithms. RF is also built on decision trees. RF uses with-replacement random samples from the training data to generate decision trees, and the results of these decision trees are averaged to get the final output. The combined output from several trees tends to smooth out the volatility between trees and improves the ability to generalize the model as a whole. One appealing aspect of RF is its ability to estimate error using out-of-bag error estimates without needing a set-aside testing dataset^46^. Like the ANN and XGB, RF also had tunable hyper-parameters optimized using the Optuna optimization framework. The list of the range and optimized hyper-parameter are provided in Table 3.

**Table 3:** Optimized values of the RF hyper-parameters.

Hyper-parameters	Range	Optimized Value
min_samples_split	2–150	17
min_samples_leaf	1–100	11
max_depth	4–100	27
n_estimators	10–2000	355

### Mapping method

After selecting the best algorithm from the four algorithms described in the previous section, we employ the best algorithm to build spatial maps. To build these maps, we select SST and SSS from different products, and the rest of the input variables are chosen from the same data used for acquiring collocated data at SOCAT cruise locations. The SST is taken from the GLORYS12V1 product, which is a CMEMS eddy-resolving reanalysis product (data available at https://data.marine.copernicus.eu/product/GLOBAL_MULTIYEAR_PHY_001_030). The SST has a spatial resolution of 1/12° and is available from 1993 to 2020. We obtained the SSS from ESA-CCI (ESA stands for European Space Agency, and CCI stands for Climate Change Initiative), a merged product of three satellite data (SMOS, Aquarius, and SMAP). This ESA-CCI (having a spatial resolution of 0.25°) is reported to perform excellently for the BoB region^47^. The ESA-CCI SSS is available at https://catalogue.ceda.ac.uk/uuid/fad2e982a59d44788eda09e3c67ed7d5.

Since ESA-CCI is available only for the period from 2010–2020, we predict sea-surface *p*CO_2_ for the previous decade (2010–2020) and then average it to form a climatology. The mean of the common period (2010–2020) is centered around 2015. Thus, 2015 is the climatological reference year for the INCOIS-ReML sea-surface *p*CO_2_ climatology. The reason for making climatology is to reduce the uncertainty caused by extreme events. All the independent data are interpolated to 1/12° resolution (same as SST, CHL, and MLD) and provided to the model for prediction. Further, we compare our product with the climatology produced by averaging *p*CO_2_ of RAMA (which is available from November 2013 to November 2018) and the gridded SOCAT data (having a spatial resolution of 1° and temporally available from 2010 to 2020). This product is expected to help in evaluating high-resolution bio-physical model simulated ocean carbon cycle as only a limited number of spatial *p*CO_2_ observations are available in the BoB across different time scales.

### CO_2_ flux calculation

After preparing the climatological sea-surface *p*CO_2_ for the BoB region, we calculate air-sea CO_2_ flux to examine the sink and source regions of the BoB. The flux is calculated using the following equation.

2 $${{\rm{CO}}}_{{\rm{2}}}\,{\rm{flux}}={\rm{kw}}\times {\rm{L}}\times \Delta {{\rm{pCO}}}_{{\rm{2}}}$$

where kw is the piston velocity calculated as a function of wind speed^48^. We use ERA5^49^ winds (https://www.ecmwf.int/en/forecasts/dataset/ecmwf-reanalysis-v5) to calculate the piston velocity^50^. L represents solubility of CO_2_^51^, and Δ *p*CO_2_ is the difference between sea-surface *p*CO_2_ and atmospheric *p*CO_2_.

## Data Records

The high-resolution sea-surface *p*CO_2_ maps and associated CO_2_ flux data produced for the BoB (reported in this paper) could be accessed from https://zenodo.org/record/8375320. ^52^ The dataset contains two products, the first being sea-surface *p*CO_2_ and the second being air-sea CO_2_ flux for the BoB region. It is a monthly climatological data. Each of these data has a spatial resolution of 1/12°. A positive value of CO_2_ flux indicates outgassing of CO_2_, and the negative value shows uptake of atmospheric CO_2_.

## Technical Validation

In this study, we use the Taylor diagram representation^53^ to evaluate the performance of the models. The Taylor diagram provides a summarized graphical view of the model performance with respect to the available observation data. Three statistics, namely Correlation Coefficient (r), Standard Deviation (STD), and Centred Root Mean Square Difference (CRMSD), are used to create the Taylor Diagram. The correlation coefficient ranges between −1 and 1; higher negative or positive values represent a strong inverse or in-sync relation between prediction and observation. Ideally, the STD of predicted values should be the same as observed, and lower CRMSD represents better model performance.

### Model selection

**Fig. 3:**
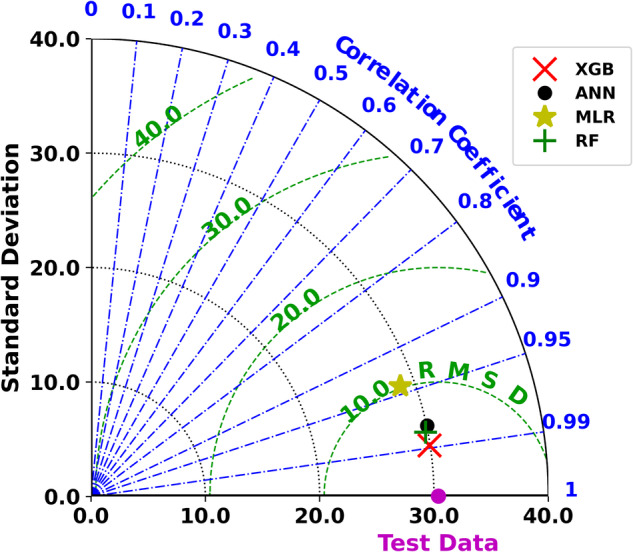
Comparison of model performance with respect to the test data.

Figure 3 represents the performance of all four models against a common test data. The performance of multiple linear regression is the worst, whereas the ANN, RF, and XGB perform almost closely to each other. The CRMSD (centered root-mean-square difference) of ANN, XGB, and RF is 6.26, 4.52, and 5.71 *μ*atm, respectively. At the same time, the correlation of ANN, XGB, and RF is, respectively, 0.978, 0.988, and 0.982. Based on the statistics, XGB seems to have a slight edge over the other two ML models. The STD of the test data is 30.38 μatm , and all three models (ANN, XGB, and RF) are very close to this STD. Hence, from Fig. 3, it is clear that the XGB performs best among the four ML models chosen in this study. Thus, we employ the XGB model to build sea-surface *p*CO_2_ maps for the BoB. Henceforth, we refer to the XGB-based climatological data product as INCOIS-ReML (Indian National Centre for Ocean Information Services-Regional Machine Learning model).

### Creating sea-surface *p*CO_2_ maps

**Fig. 4:**
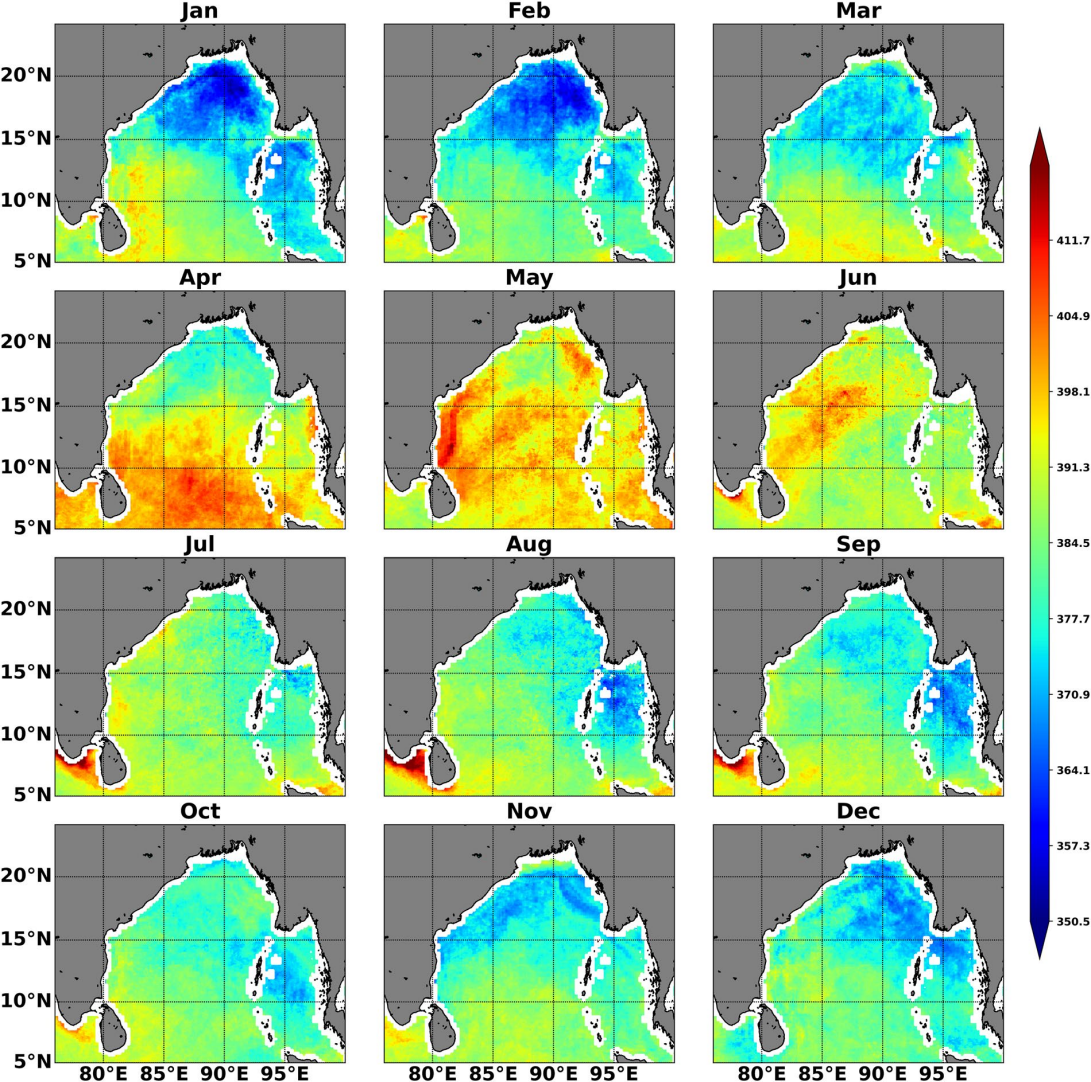
Climatological monthly variability of the sea-surface *p*CO_2_ produced by INCOIS-ReML. The climatological reference year for this dataset is 2015.

**Fig. 5:**
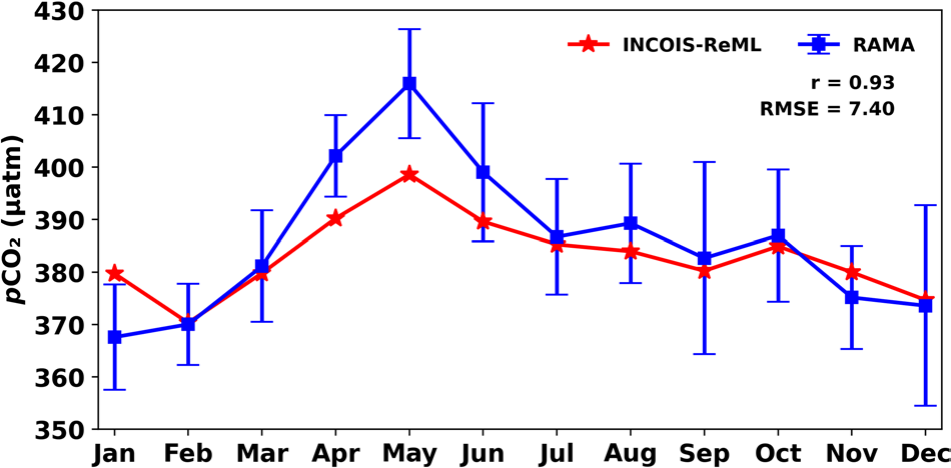
Climatological monthly variability of the sea-surface *p*CO_2_ produced by INCOIS-ReML is compared with the climatology created by RAMA mooring buoy. The climatological reference year for this dataset is 2015.

INCOIS-ReML is a high-resolution monthly climatological data product (Fig. 4). The temporal evolution of the INCOIS-ReML *p*CO_2_ climatology has been compared with BOBOA mooring-based *p*CO_2_ climatology (averaging over the available observation from 2014–2018) using correlation, root mean square error (RMSE), and Willmott skill score (WSS)^54^. The monthly variability of sea-surface *p*CO_2_ is satisfactorily captured by the INCOIS-ReML (correlation (r) = 0.93; Fig. 5). This comparison shows that INCOIS-ReML underestimates the sea-surface *p*CO_2_ (particularly in April and May). However, the RMSE between the observed and modeled values is 7.40, which indicates that the error is within acceptable bounds (Fig. 5). The capability of INCOIS-ReML *p*CO_2_ is also evident from its WSS of 0.885.

**Fig. 6:**
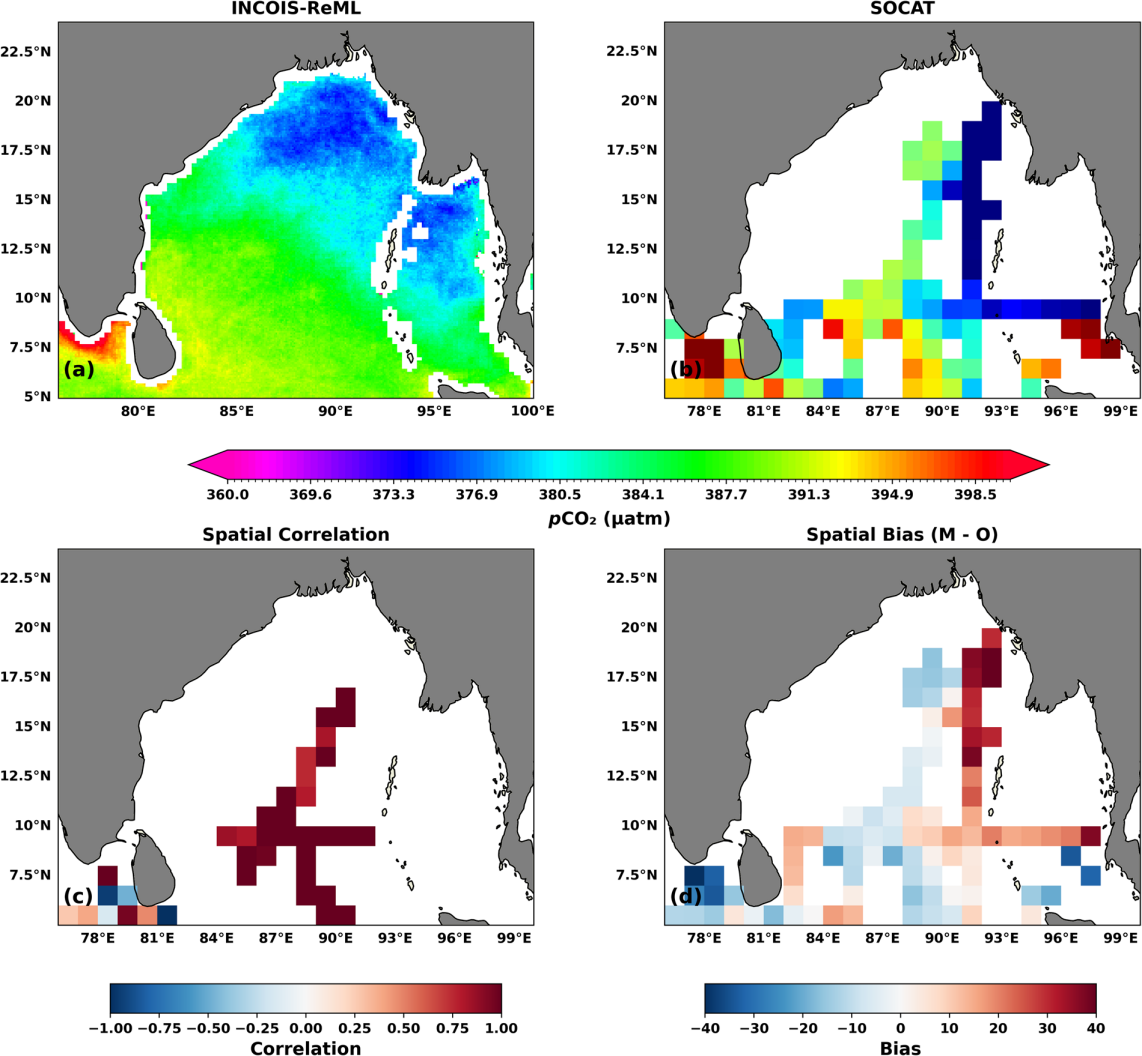
Comparison (annual mean of the climatological year) between the (**a**) INCOIS-ReML produced sea-surface *p*CO_2_ and (**b**) SOCAT. The spatial correlation and spatial bias (difference (Model - Observation (M-O)) in an annual mean of the climatological year) are shown in figures (**c**) and (**d**). The climatological reference year for this dataset is 2015.

Using the available observations from BOBOA mooring (location-specific data), we validated the temporal variability of INCOIS-ReML *p*CO_2_. However, a limited number of observations makes it difficult to validate spatial variability of INCOIS-ReML *p*CO. Therefore, we use observations-based gridded (1° × 1°) SOCAT product (available from the 1990s to date) to compare spatial variability of *p*CO_2_. As a first step, we generate a climatology of SOCAT data product with reference to the year 2015 for comparison. Before comparison, we interpolate the high-resolution INCOIS-ReML data product (Fig. 6a) to the spatial resolution of SOCAT gridded data product (Fig. 6b) using the nearest-neighbor interpolation method. Here, the reader must understand that the unavailability of a sufficient number of temporally varying observations in the BoB impacts the magnitude of the sea-surface *p*CO_2_ climatology derived from SOCAT. INCOIS-ReML satisfactorily captures the spatial pattern, i.e., lower sea-surface *p*CO_2_ in the north and higher sea-surface *p*CO_2_ in the south. Figure 6c,d provide spatial statistics to evaluate the performance of the INCOIS-ReML data product. A high correlation is seen in the central BoB region (Fig. 6c). A few grids show negative to low correlation in the south of the Sri Lankan coast. Figure 6d shows overestimation in the region east of 92° E, but low negative bias persists in the rest of the region. The domain average bias is approximately 0.92 *μ*atm. The overestimation of the INCOIS-ReML can be attributed to the discontinuous time-series data from SOCAT in a large part of BoB.

We compare the INCOIS-ReML *p*CO_2_ with the results of existing studies, carried out using *in-situ* observations, available in the literature to validate the spatial variability of *p*CO_2_ more rigorously. The spatial monthly variation of INCOIS-ReML is shown in Fig. 4. The northern BoB (approximately above 15° N) is seen to have lower sea-surface *p*CO_2_ than the southern BoB region^55,56^. The EICC (East India Coastal Current) is known to have dominant control over the sea-surface *p*CO_2_, especially in the western coast of BoB^16^ due to the spreading of river-influenced water along the coast. The northward-moving EICC is primarily strong from March to May when high salinity and *p*CO_2_ levels are observed. In contrast, southward-moving EICC during October to December brings river-influenced low saline and *p*CO_2_ water along the coast^16^. The INCOIS-ReML well reproduces the coastal pattern of *p*CO_2_ levels with the lowest during November and the highest *p*CO_2_ levels during May (Fig. 4). Overall, the spatial and temporal patterns are well captured by INCOIS-ReML.

**Fig. 7:**
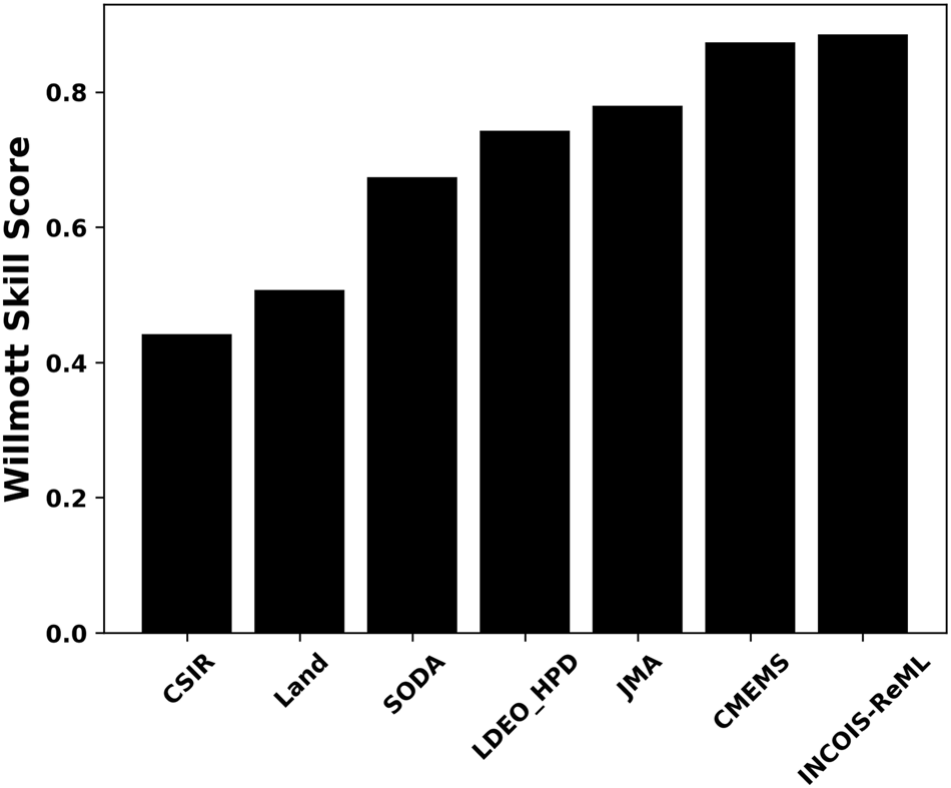
Willmott Skill Score of the comparison between INCOIS-ReML and other six widely used ML-based products' climatological *p*CO_2_ with BOBOA based climatological *p*CO_2_ observations.

**Fig. 8:**
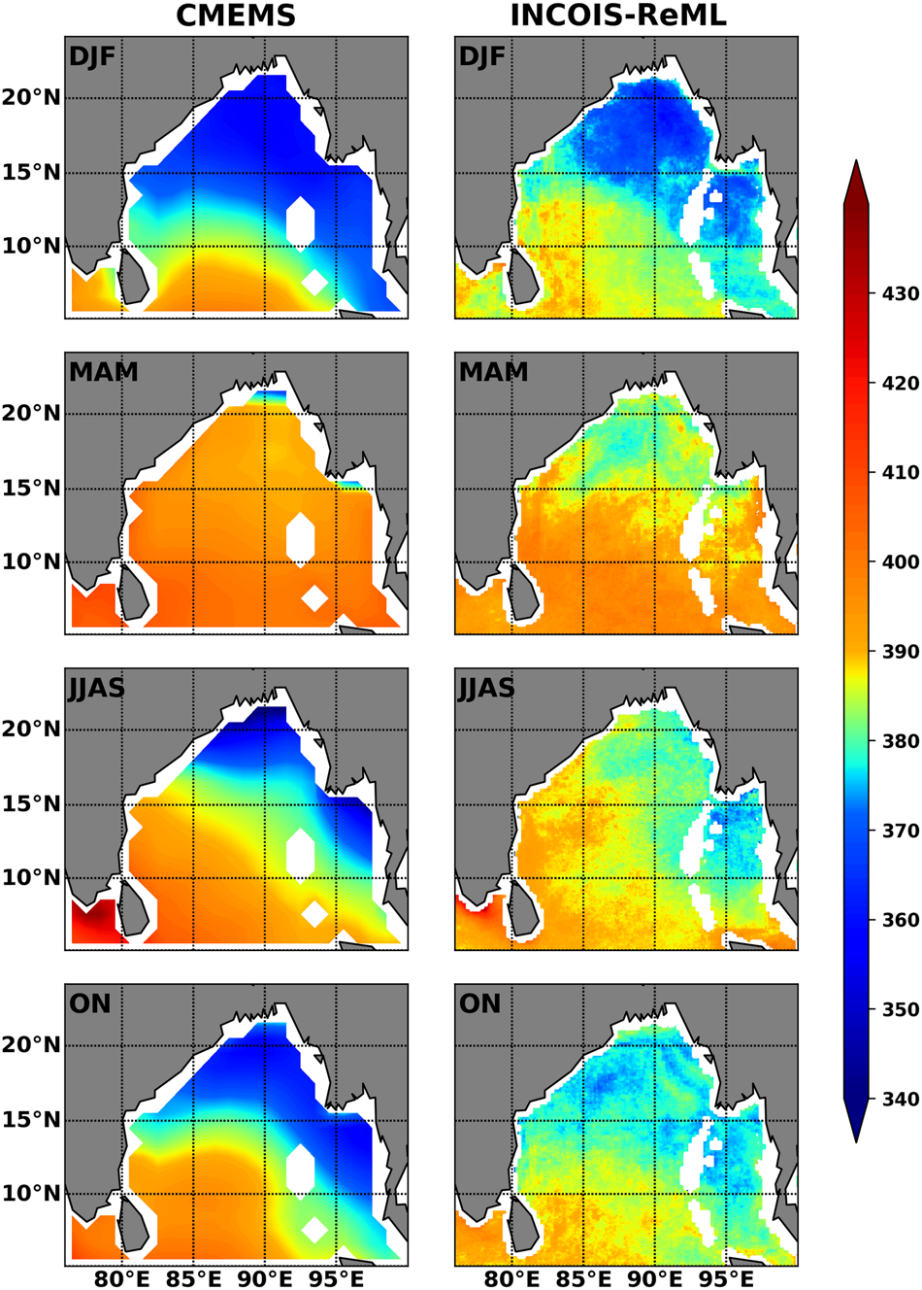
Seasonal spatial comparison of *p*CO_2_ between CMEMS and INCOIS-ReML.

Further, we compare our climatological product with six widely used ML-based *p*CO_2_ products (listed in Table 4). Figure 7 shows that based on the Willmott skill score (WSS), INCOIS-ReML performs better than all the other six products. This is due to two primary reasons: a) the inclusion of a significant number of open and coastal ocean observations from SAS leads to an improvement in model prediction, and b) the high spatial resolution of INCOIS-ReML. Figure 7 (based on WSS) shows that CMEMS performs as good as INCOIS-ReML. Hence, we further compare the two products spatially and explain the advantages of high-resolution INCOIS-ReML (Fig. 8).

**Table 4:** List of ML-based models with which we compare INCOIS-ReML.

Abbreviations	Full Form
CMEMS	CMEMS-LSCE-FFNN^25^
LAND	SOMFNN^62^
SODA	OceanSODAETHZ^63^
LDEO_HPD	SpCO_2__LDEO_HPD^64^
JMA	JMAMLR^65^
CSIR	CSIRML6^26^

The first observation from Fig. 8 is that INCOIS-ReML is capable of capturing spatio-temporal variability of *p*CO_2_ in the coastal waters of the BoB. Since BoB receives high freshwater flux from rivers and precipitation during the southwest monsoon (June-September), low salinity water is found in the north that spreads to the south by monsoon currents^57^. This freshwater plume spreads to the BoB by fall monsoon (ON)0^15,58,59^. This plume first spreads in the eastern Bay, followed by the western Bay, with minimal impact on freshwater during spring inter-monsoon (March to May). CMEMS and INCOIS-ReML performed well in capturing spatial variations of low *p*CO_2_ primarily driven by the low saline waters in the BoB. However, the spatial variations were not well captured by CMEMS compared to INCOIS-ReML during spring monsoon (MAM). Perennial occurrence of low *p*CO_2_ due to low salinity during the summer monsoon season was reported in the northern BoB^60^, that was not well captured by CMEMS (Fig. 8). In addition, the *p*CO_2_ levels in the low salinity plume region were underestimated by CMEMS compared to INCOIS-ReML^61^. The presence of low-saline freshwater and associated strong stratification lower the sea-surface *p*CO_2_ values in the northern BoB^16^. These physical processes play a significant role in regulating the seasonality of sea-surface *p*CO_2_ in the BoB^15,17,19^. It is evident that the seasonality of sea-surface *p*CO_2_ is well captured by the INCOIS-ReML. Therefore, the high resolution INCOIS-ReML data product is an improved version of the climatological mean state of sea-surface *p*CO_2_ in the BoB region.

Hence, we provide a high-resolution sea-surface *p*CO_2_ maps and associated air-sea CO_2_ flux (calculated using the equation mentioned in the earlier section) data product, which would immensely aid in validating not only high-resolution bio-physical model simulated ocean carbon cycle but also coarser-resolution CMIP6 models. Further, it is worth mentioning that the inclusion of SAS data makes it possible for this high-resolution product to capture the coastal *p*CO_2_ dynamics better, which is missing in other observation-based data products. We understand that the product can still be improved, and we will keep on updating the product as the number of observations increases. This product is expected to be extremely helpful in validating models (especially spatial variability) used to understand the future scenarios of the sea-surface *p*CO_2_ in the BoB.

## Data Availability

The code used to create the final product (different machine learning models) is available at https://github.com/APJ1812/INCOIS_pCO2. The study uses general machine learning codes available in Python.
